# Thymidine Kinase 1 Drives Skin Cutaneous Melanoma Malignant Progression and Metabolic Reprogramming

**DOI:** 10.3389/fonc.2022.802807

**Published:** 2022-03-03

**Authors:** Sipeng Zuo, Huixue Wang, Lin Li, Hui Pan, Linna Lu

**Affiliations:** ^1^Department of Ophthalmology, Ninth People’s Hospital, Shanghai Jiao Tong University School of Medicine, Shanghai, China; ^2^Shanghai Key Laboratory of Orbital Diseases and Ocular Oncology, Shanghai, China

**Keywords:** skin cutaneous melanoma, thymidine kinase 1, tumorigenesis, cell metabolism, bioinformatics

## Abstract

**Background:**

Thymidine kinase 1 (TK1) is a cell cycle-dependent kinase that catalyzes the addition of a gamma-phosphate group to thymidine. The protumorigenic role of TK1 has been reported in various malignancies. However, the role of TK1 in skin cutaneous melanoma (SKCM) remains unclear. This study aimed to explore the molecular function of TK1 in SKCM progression.

**Methods:**

Bioinformatics data were acquired from The Cancer Genome Atlas (TCGA) and Gene Expression Omnibus (GEO). Subcutaneous xenografts were established to observe the effect of TK1 knockdown on the proliferation of SKCM cells *in vivo*. RNA sequencing (RNA-seq; deposited in Sequence Read Archive, SRX10950283-SRX10950285 for A375 control cells and SRX10950286-SRX10950288 for TK1-silenced A375 cells) and immunoprecipitation–mass spectrometry (IP-MS) were used to analyze TK1-related genes and pathways. Seahorse XF Cell Mito tests and glycolysis stress assays were conducted for metabolic testing.

**Results:**

TK1 was upregulated in malignant SKCM compared to that in normal tissues and cell lines. Elevated expression of TK1 was associated with poor prognosis. *In vitro* and *in vivo* assays demonstrated that TK1 promoted the proliferation and migration of SKCM cells. Moreover, TK1 was strongly associated with multiple intracellular metabolic pathways, facilitating cell mitochondrial respiration and glycolysis in SKCM malignant progression.

**Conclusions:**

TK1 drives SKCM malignant progression and supports metabolic reprogramming, indicating that TK1 serves as a therapeutic target for SKCM.

## Introduction

Skin cutaneous melanoma (SKCM) is a common cutaneous cancer with an unfavorable prognosis. To date, over 160,000 new cases have been reported annually, including 48,000 deaths (1, 2). Most SKCMs result from ultraviolet radiation from sunlight (3). In particular, Caucasian descent, light-colored eyes, and family history are considered to be risk factors for SKCM (4). SKCM displays a high tendency to invade and metastasize, frequently to the distant organs including the lungs, liver, and brain (2). More importantly, the prognosis for most advanced SKCM cases is unfavorable because of the development of drug resistance to common chemotherapies (5). Therefore, further investigation of the mechanism underlying SKCM tumorigenesis and progression could improve outcomes of melanoma patients.

Recently, there has been an increased understanding of SKCM malignant progression, especially in terms of immunotherapy targeting immune checkpoints (6, 7). Immune checkpoint inhibitors, particularly anti-Cytotoxic T lymphocyte-associatedantigen-4 (CTLA4) and anti-Programmed cell death protein-1 (PD-1) antibodies, have revolutionized the treatment of many cancers, including advanced melanomas (7). Moreover, combination regimens of immune checkpoint inhibitors and other small-molecule inhibitors, such as those targeting the Ras/Raf/Mitogen-activated protein/extracellular signal-regulated kinase (ERK) kinase (MEK) pathway, have achieved better inhibitory efficacy in treating SKCM (8, 9). However, recalcitrance to cytotoxic chemotherapy and limited treatment options remain major challenges for treating SKCM (10). Based on the considerable threat of SKCM, it is necessary to explore additional therapeutic targets.

Thymidine kinase 1 (TK1) is a cell cycle-dependent phosphorylase that is located in the cytoplasm. Emerging evidence has shown that TK1 is highly expressed across diverse cancers. TK1 is associated with DNA replication and cell proliferation by converting thymidine to thymidine monophosphate (11). TK1 is associated with accelerated cell cycle transition in tumors. Normally, TK1 expression increases in the G1/S transition and reaches the summit in the S phase, whereas it remains limited in the rest of the cell cycle (12). Moreover, TK1 acts as a predictive marker for several cancer types, including breast cancer and gastrointestinal cancer (13, 14). In addition, TK1 has been applied in cancer screening and chemotherapy monitoring in several clinical practices (15–18). However, the biological function of TK1 in SKCM remains to be elucidated.

To further illustrate the role of TK1 in SKCM, we uncovered the expression patterns of TK1 in SKCM in both clinical samples and cell lines. Functional assays revealed that TK1 acts as an oncogene in SKCM both *in vitro* and *in vivo*. Moreover, we revealed for the first time that TK1 is associated with metabolic reprogramming. In conclusion, our study revealed that TK1 functions as a novel anticancer target for treating SKCM.

## Materials and Methods

### Cell Culture

PIG1 (normal human melanocyte cell line) and SK-MEL-28 (human SKCM) cells were cultured in RPMI 1640 medium (Gibco). The human SKCM cell lines A375, SK-MEL-5, LOX-IMVI, M14, and M21 were cultured in Dulbecco’s modified Eagle’s medium (Gibco). All media were supplemented with 10% fetal bovine serum (FBS; Gibco), 100 U/ml penicillin, and 100 mg/ml streptomycin, and all cell lines were cultured in a humidified atmosphere containing 5% CO_2_ at 37°C. The medium was refreshed every 2 days. The PIG1 normal human melanocyte cell line was kindly provided by the Department of Ophthalmology, Peking University Third Hospital. The A375, SK-MEL-5, SK-MEL-28, M14, and M21 cell lines were purchased from the American Type Culture Collection (ATCC, https://www.atcc.org/). LOX-IMVI melanoma cells were purchased from Millipore Sigma.

### RNA Isolation and Quantitative Real-Time PCR

Total RNA was harvested from cells using TRIzol reagent (Invitrogen) according to the standard manufacturer’s protocol. RNA was then reverse-transcribed using the PrimeScript RT Reagent Kit with gDNA Eraser (Takara). Quantitative real-time PCR (qRT-PCR) analyses were performed using Power SYBR Green PCR Master Mix (ABI) on a Roche LightCycler480 System. The glyceraldehyde-3-phosphate dehydrogenase (*GAPDH*) gene served as a reference control. Each experiment was performed in triplicate. The primer sets used are listed in **Supplementary Table S1**.

### Western Blot Analysis

Cells were harvested at the indicated times and rinsed three times with PBS. Cell extracts were prepared with a lysis buffer and centrifuged at 13,000 × g for 30 min at 4°C. Protein samples were separated by 7.5% (w/v) sodium dodecyl sulfate–polyacrylamide gel electrophoresis (SDS-PAGE) and transferred to polyvinylidene fluoride membranes. After blocking with 5% milk for 1 h at room temperature, the membrane was incubated with 2.5 μg/ml of antibody in 5% Bovine serum albumin (BSA) overnight at 4°C with specific primary antibodies including anti-TK1 (1:1,000 dilution, Abcam), anti-E2F1 (1:2,000 dilution, Proteintech), anti-CCNB1 (1:2,000 dilution, Proteintech), anti-CDK2 (1:2,000 dilution, Proteintech), anti-GAPDH (1:5,000 dilution, Proteintech), or anti-β-actin (1:5,000 dilution, Proteintech). After washing with a Tris-buffered saline with Tween solution (TBST, 50 mmol/L Tris-HCl, pH 8.0, 150 mmol/L NaCl, and 0.1% Tween-20) three times, the membranes were incubated with the secondary antibodies goat anti-rabbit immunoglobulin G (heavy chain + light chain)-horseradish peroxidase [IgG (H + L)-HRP, 1:10,000, Proteintech) or goat anti-mouse IgG (H + L)-HRP (1:10,000, Proteintech) for 2 h. Immunoreactive bands were identified using an enhanced chemiluminescence (ECL) kit (Millipore) and visualized with the ChemiDoc XRS system (Bio-Rad). Protein expression levels were normalized to those of total *GAPDH* or β-actin.

### Lentivirus Packaging and Generation of Stable Cell Lines

pLKO.1, pCDH, and pCMV were used in the present study. ShRNA sequences were generated by PCR and cloned into the pLKO.1 vector and verified by DNA sequencing. The Lipofectamine 3000 reagent (Invitrogen) incubated with Opti-MEM I Reduced Serum Medium (Gibco) was used to transfect 293T cells with 3.0 mg of the pMD2.D plasmid and 6.0 mg of the PsPax plasmid. Six hours after transfection, the medium was replaced with approximately 10 ml of fresh medium. The supernatants containing viruses were collected at 48 or 72 h. The virus-containing solution was filtered through a 0.45-μm cellulose acetate filter and concentrated with an Amicon Ultra-15 Centrifugal Filter Unit (Millipore) at 3,000 rpm and 4°C for 30 min, aliquoted, and frozen at -80°C for long-term storage. Twenty-four hours prior to transduction, tumor cells were seeded at 5 × 10^5^ cells per well in a 6-well plate. The medium was replaced with a virus-containing supernatant supplemented with 10 ng/ml polybrene (Sigma-Aldrich). After 48 h, the medium was replaced with fresh medium (2.5 ml). The selection was performed by incubating with 4 μg/ml puromycin (InvivoGen) for 2 weeks. Colonies were selected and expanded for further analyses.

### Immunohistochemistry Staining for Thymidine Kinase 1

Formalin-fixed and paraffin-embedded tissues were sliced into 5-μm thickness, deparaffinized with xylene, and gradually rehydrated in grated ethanol solutions. Next, the slides were incubated with the anti-TK1 antibody at 4°C overnight, followed by incubation with biotinylated goat anti-rabbit serum streptavidin–peroxidase conjugate at room temperature for 30 min. Finally, sections were developed with diaminobenzidine and counterstained with hematoxylin. Two independent pathologists who were blinded assessed the positivity and intensity of the tissue sections. The percentage of TK1-positive staining cells was scored as 0 (0%), 1 (1%), 2 (2%) … 99 (99%), 100 (100%). The staining intensity was given 0 (none), 1 (low), 2 (medium), and 3 (high) scores. A combination of the two scores was calculated by the following formula for all cases: Immunohistochemistry (IHC) score = positive rate score × intensity score.

### Colony Formation Assay

Human SKCM cells were seeded at a density of 1 × 10^3^ cells/well in 6-well plates. Cells were cultured for 2 weeks in respective complete media in a 37°C humidified atmosphere of 5% CO_2_. The colonies were then stained with crystal violet and photographed.

### Cell Counting Kit-8 and Transwell Assay

The cells were seeded into 96-well plates at a density of 3,000 cells per well. Here, 10 μl of Cell Counting Kit-8 (CCK-8) reagent (Dojindo) and 100 μl complete medium were mixed and added to each well to detect cell viability. After incubation in the dark at 37°C for 3 h, the absorbance of each well was measured at 450 nm. For migration assays, 2 × 10^4^ cells in 200 μl of 2% FBS medium were seeded in the Transwell upper chambers, and the lower chambers were filled with 10% FBS medium. After 24 h, the cells remaining in the upper Transwell chamber were removed, and those that migrated to the lower chamber were photographed and counted.

### Wound Healing Assay

SKCM cells were seeded onto 6-well plates and grown to more than 90% confluence and then wounded using a sterilized pipette tip to make a straight scratch. The cells were then washed twice with fresh culture medium and cultured for another 24 h, and images were taken at 0 and 24 h using an Olympus digital camera. The distance of wound healing was measured with ImageJ 1.46r software. Each experiment was performed in triplicate.

### Immunofluorescence

SKCM tumor tissues were fixed in 4% formalin and paraffin-embedded. The specimens were cut into 4-µm-thick sections and then fixed with 4% paraformaldehyde fixative at room temperature for 20 min and washed in PBS three times. The sections were then incubated with a blocking solution (normal goat serum) at room temperature for another 20 min. Next, the samples were incubated with anti-TK1 antibody overnight at 4°C. After being washed in PBS three times, the sections were incubated with secondary antibodies for 1 h at room temperature. Finally, coverslip-mounted slides were observed and photographed under a fluorescence microscope.

### Subcutaneous Xenograft

Animal ethics were approved by the Institutional Animal Care and Use Committee of the Ninth People’s Hospital, Shanghai Jiao Tong University School of Medicine. Eighteen 5‐week‐old BALB/c nude mice were obtained from the Model Animal Research Center of Shanghai Jiao Tong University School of Medicine. To evaluate the proliferative function of TK1, approximately 2 × 10^6^ melanoma cells from each group were injected subcutaneously into the right side of the abdomen of BALB/c nude mice (female, 5 weeks old). Tumor volume was measured every 3 days. After 3 weeks of tumor cell injection, the nude mice were sacrificed, and the tumors were excised and photographed. Tumor volume = (short diameter)^2^ × (long diameter)/2.

### RNA Extraction, Library Construction, and Illumina Sequencing (RNA Sequencing)

Total RNA was extracted from A375-shTK1/shCtrl cells using the TRIzol reagent (Invitrogen). RNA integrity was confirmed using a 2100 Bioanalyzer (Agilent Technologies), and the RNA concentration was measured using a Qubit 2.0 fluorometer with a Qubit RNA Assay Kit (Life Technologies). Sequencing was performed using the Illumina HiSeq 2500 platform. Differentially expressed genes (DEGs) were then identified through fold change, and p-values were calculated using a t-test. The threshold set for upregulated and downregulated genes was fold change >1.0 or <-1.0 and p < 0.05. The raw sequence data reported in this study have been uploaded to the Sequence Read Archive in NCBI under the accession number SRX10950283-SRX10950288.

### Immunoprecipitation and Mass Spectrometry

Cells were lysed with immunoprecipitation (IP) lysis buffer (20 mM Tris-HCl, pH 7.4; 150 mM NaCl; 1 mM EDTA; and 1% NP-40 lysis). After high-speed centrifugation at 4°C, the supernatant was incubated with Protein G Sepharose beads and the indicated antibody overnight at 4°C. The mixture was then centrifuged at 3,000 rpm at 4°C. The beads were washed three times with buffer containing 300 mM and 150 mM NaCl and resuspend in 2× SDS loading buffer, incubated at 95°C for 10 min, and finally analyzed by Western blotting (WB). For immunoprecipitation–mass spectrometry (IP-MS) analysis, the supernatants were separated using SDS-PAGE gels. To perform liquid chromatography–tandem mass spectrometry (LC-MS/MS) analysis, the digestion products were separated through 120-min gradient elution at a flow rate of 300 nl/min in an Easy-nLC 1000 directly interfaced with a Thermo Q Exactive HF mass spectrometer. The MS/MS spectra from each LC-MS/MS run were subjected to searches against the selected database using MaxQuant1.6.0.1.

### Cell Metabolism Tests

Mitochondrial oxidative phosphorylation (OxPhos) and glycolysis levels were determined and analyzed using an Agilent Seahorse Bioscience XF96 Analyzer (Agilent Technologies) according to manufacturer protocols. Specifically, melanoma cells (3 × 10^4^) were seeded into each well of the cell culture microplates. For the Mito stress assay, cells were sequentially treated with oligomycin (10^-3^ mM), Trifluoromethoxy carbonylcyanide phenylhydrazone (FCCP) (10^-3^ mM), and rotenone/antimycin A (5 × 10^-4^ mM). The oxygen consumption rate (OCR; pmol/min) was recorded using an Agilent Seahorse Bioscience XF96 analyzer. For the glycolysis stress assay, cells were exposed to glucose (10 mM), oligomycin (10^-3^ mM), and 2-deoxy-D-glucose (2-DG; 50 mM), and then the extracellular acidification rate (ECAR; mpH/min) was recorded using an Agilent Seahorse Bioscience XF96 Analyzer. To exclude the influence of proliferation difference of different cell lines on measurement results, the same batch of cells was simultaneously plated on 24-well plates (80,000 cells per well) in the same operation on the day before the experiment and incubated overnight. On the day of the experiment and the day after the experiment, cells in 24-well plates were lysed with SDS lysate, and protein content was determined using a Bicinchoninic acid (BCA) kit. The protein ratio was used to represent the number difference after cell proliferation and for normalization.

### Statistical Analysis and Bioinformation

Experiments were performed three times *in vitro*. All data were presented as mean ± standard deviation (SD). Difference analyses were performed using an unpaired Student’s t-test in Prism 7.0 software (GraphPad) and ∗p < 0.05, ∗∗p < 0.01, ∗∗∗p < 0.001, ∗∗∗∗p < 0.0001. Statistical analysis was performed using SPSS version 20.0. TK1 was searched on http://www.cbioportal.org/ and TCGA to explore the expression or variant differences and conduct pan-Cancer Atlas and Gene Ontology (GO) analyses. Datasets GSE46517, GSE19234, and GSE22155 from the GEO database were collected and analyzed. Kaplan–Meier analysis was used to investigate the expression of TK1 and its association with prognosis. A significant difference was considered statistically with p-value <0.05.

## Results

### Increased Thymidine Kinase 1 Refers to Unfavorable Outcome in Skin Cutaneous Melanoma

**Figure 1:**
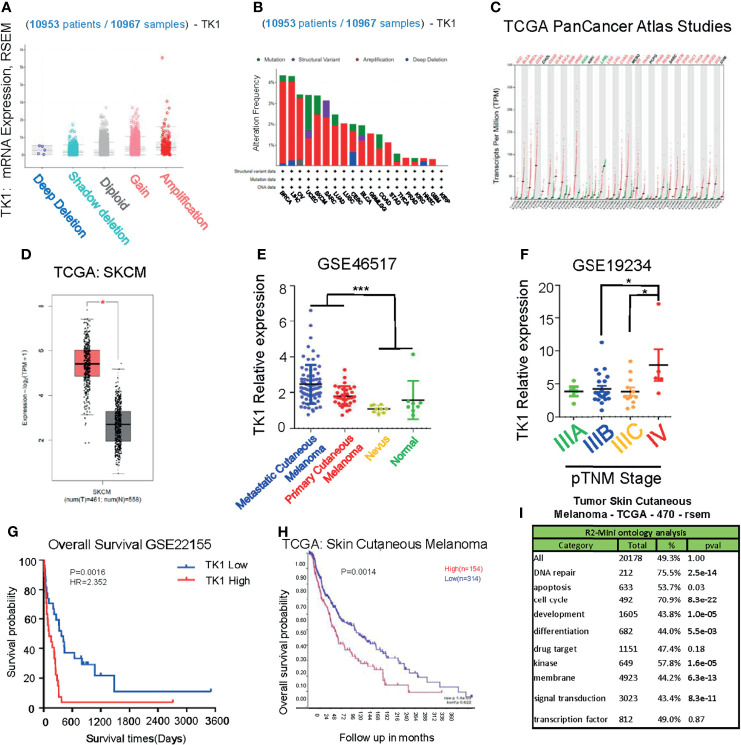
Upregulation of thymidine kinase 1 (TK1) in skin cutaneous melanoma (SKCM) in databases. **(A)** Pan-Cancer analysis of TK1 in The Cancer Genome Atlas (TCGA) database. **(B)** Structural variants of TK1 in diversified tumors in 10,953 patients/10,967 samples. **(C)** The elevated TK1 level in 25 kinds of malignancies. **(D, E)** Relative TK1 expression in normal and tumor tissues. The tumor tissues were 461, and the normal tissues were 558. **(F)** Relative TK1 expression in different pTNM stages from IIIA to IV. **(G, H)** Unfavorable prognosis due to higher TK1 levels in Gene Expression Omnibus (GEO) and TCGA cohorts. **(I)** Gene Ontology (GO) analysis to reveal TK1 function in cancer. *p < 0.05, ***p < 0.001.

To investigate the potential role of TK1 in tumorigenesis, we performed a pan-cancer analysis of TK1 in TCGA database, which included 10,953 participants with 10,967 malignancies (**Figure 1A**) using cbioportal. We found that TK1 presented with frequent structural variants, and copy number amplification indicated enhanced TK1 expression in diverse tumors, including breast invasive carcinoma, liver hepatocellular carcinoma, ovarian serous cystadenocarcinoma, uterine corpus endometrial carcinoma, and SKCM (**Figure 1B**). More importantly, the majority of malignancies (25 of 33 types of cancer, 75.8%) presented with upregulated TK1 levels (**Figure 1C**). Among these TK1-upregulated tumors, the role of TK1 in SKCM remains to be fully addressed. To further investigate the expression of TK1 in SKCM, data from GEO and TCGA were acquired for analysis. SKCM tissues had higher TK1 levels than normal tissues (**Figure 1D**; p < 0.05). In the meantime, an overall difference in the expression of TK1 between the primary or metastatic SKCM and nevus or normal tissue was shown (**Figure 1E**; p = 0.0003). Moreover, TK1 expression was higher in advanced patients than that in those with early cutaneous melanoma (**Figure 1F**; p < 0.05). In addition, based on the data provided by GEO and TCGA, we found that TK1 expression was associated with an unfavorable outcome, both in GEO (**Figure 1G**; p < 0.01) and TCGA cohorts (**Figure 1H**; p<0.01). Most importantly, GO analysis revealed that TK1 expression was related to several important oncogenic/tumor suppressor pathways, including DNA repair (75.5%, p = 2.5e-14), apoptosis (53.7%, p = 0.03), and cell cycle (70.9%, p = 8.3e-22; **Figure 1I**).

### Thymidine Kinase 1 Supported Proliferation and Migration of Skin Cutaneous Melanoma Cells *In Vitro*

**Figure 2:**
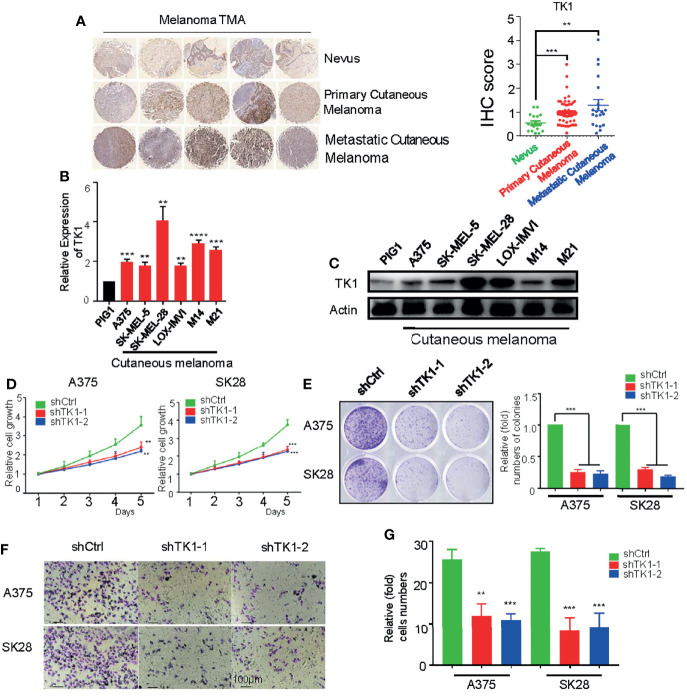
A protumor role of thymidine kinase 1 (TK1) was confirmed in skin cutaneous melanoma cell lines. **(A)** Immunohistochemistry (IHC) staining on dozens of nevi, primary skin cutaneous melanomas (SKCMs), and metastatic SKCMs with respective fold change of TK1. **(B)** Relative mRNA expression of TK1 in different melanoma cell lines by quantitative real-time PCR (qRT-PCR). The experiments were performed in triplicate. **(C)** Relative protein expression of TK1 in different melanoma cell lines by Western blot. The experiments were performed in triplicate. **(D, E)** Cell Counting Kit-8 (CCK-8) assay and colony formation assay were conducted to confirm the influence of TK1 on cell proliferation. The number at the first day was set to 1 for every group. The experiments were performed in triplicate. **(F, G)** Transwell assay and statistics were analyzed to explore the influence of TK1 on SKCM cell migration. The experiments were performed in triplicate. **p < 0.01, ***p < 0.001.

To investigate TK1 expression levels in SKCM, we conducted IHC (**Figure 2A****, left**) staining and immunofluorescence (IF) (**Supplementary Figure S1**) assays of nevi (normal control), primary SKCMs, as well as metastatic SKCMs. Herein, we found that TK1 was remarkably increased in both primary and metastatic SKCMs (**Figure 2A****, right**). Notably, compared to primary melanoma, metastatic SKCM presented with an elevated trend of TK1 expression; however, the difference did not reach statistical significance, as measured by both IHC (p = 0.0902) and IF (p = 0.2199). Conclusively, these results demonstrated that TK1 expression was significantly increased in both primary SKCM and metastatic SKCM; however, no statistical change of TK1 expression was observed between primary and metastatic SKCMs. In addition, mRNA and protein levels in SKCM were higher than those in normal pigment cells (PIG1) (**Figures 2B, C**), indicating that TK1 may function as a diagnostic marker for SKCM.

To explore the oncogenic role of TK1 in SKCM, two SKCM cell lines (A375 and SK-MEL-28) were lentivirally transduced with shRNA against TK1. As expected, the expression of TK1 was significantly decreased in TK1-silenced cells (**Supplementary Figure S2**). Moreover, cell growth was significantly inhibited after TK1 silencing (**Figure 2D**). More importantly, there were fewer and smaller colonies in the TK1-inhibited group (**Figure 2E**). To study the influence of TK1 knockdown on migration, a Transwell assay was performed. Notably, the migration ability was remarkably decreased after knockdown of TK1 (**Figures 2F, G**; **Supplementary Figure S3**). These results indicate that TK1 plays a vital role in the proliferation and migration of SKCMs.

### Thymidine Kinase 1 Silencing Inhibited Skin Cutaneous Melanoma Growth *In Vivo*

**Figure 3:**
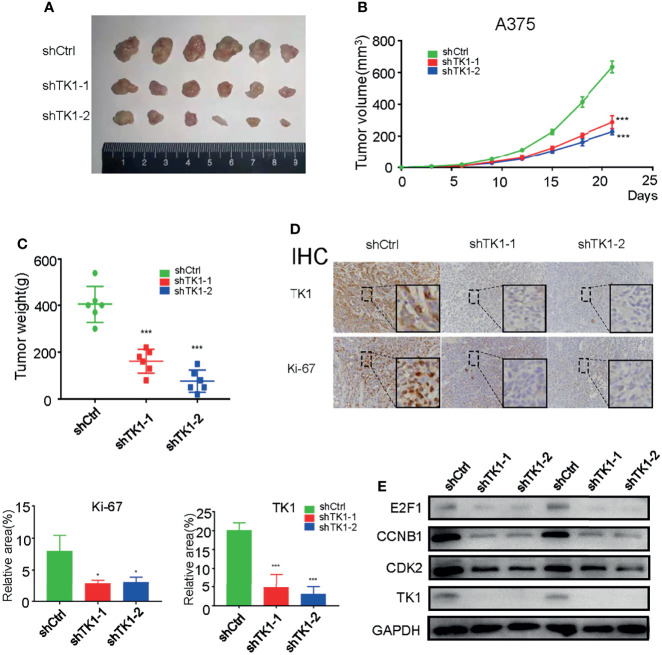
Thymidine kinase 1 (TK1) silencing reduced skin cutaneous melanoma (SKCM) oncogenic progression *in vivo*. **(A)** Tumors excised from nude mice after 3 weeks. **(B, C)** Tumor volumes and weights. **(D)** Ki-67 and TK1 index of tumors in the control group and TK1 knockdown group. **(E)** Downstream effectors of the TK1 pathway from tumor tissues were downregulated. *p < 0.05, ***p < 0.001.

To further confirm the effect of TK1 knockdown on SKCM cell proliferation *in vivo*, a xenograft model of subcutaneous tumors in nude mice was used. Subcutaneous tumors were harvested after 3 weeks (**Figure 3A**). Importantly, the volume and weight of tumors were reduced in the TK1 knockdown group (**Figures 3B, C**). In addition, decreased expression of Ki-67 was also observed in the TK1 knockdown group, which indicated that the cells retained a less active status of proliferation in TK1-silenced xenografts (**Figure 3D**). Previous studies have indicated that *E2F1*, *CCNB1*, and *CDK2* serve as downstream effectors for TK1 (19, 20). Consistently, we also observed decreased expression of these downstream targets after inhibiting TK1 expression (**Figure 3E**). These results suggest that TK1 silencing impairs SKCM proliferation *in vivo*.

### Thymidine Kinase 1 Was Associated With Metabolism Reprogramming in Skin Cutaneous Melanoma

**Figure 4:**
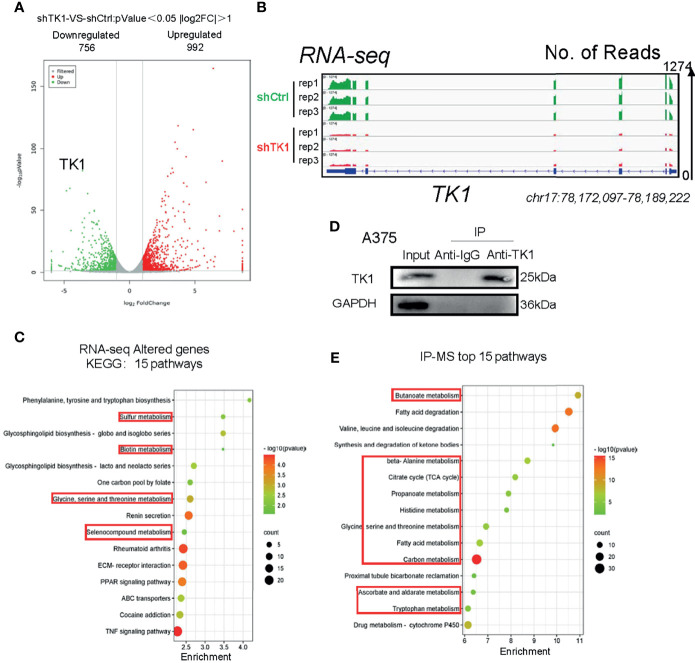
Thymidine kinase 1 (TK1) was associated with skin cutaneous melanoma (SKCM) cell metabolism disturbance. **(A)** Altered genes with TK1 knockdown in SKCM. |log2FC| >1, p < 0.05. **(B)** Integrative Genomics Viewer (IGV) shows the expression of TK1 in knockdown/control group. The data range was set to 1,274. **(C)** Fifteen pathways of top enrichment of changed genes with TK1 knockdown. **(D)** Western blot confirmed that TK1 was purified successfully in lysate and had no impurity. **(E)** Fifteen top enrichment pathways of all genes that corresponded to proteins interacting with TK1; most were relevant to intracellular metabolic processes.

Since TK1 plays an important role in the aggressive proliferation and migration of SKCM, we were interested in exploring the mechanism underlying TK1 silencing-induced SKCM inhibition. We observed a dramatic transcriptional change in TK1-silenced cells. More specifically, 992 genes were upregulated and 756 genes were downregulated (**Figure 4A**; |log2FC| >1, p < 0.05). As expected, the expression of TK1 in the knockdown group was significantly lower after TK1 inhibition (**Figure 4B**). In the Kyoto Encyclopedia of Genes and Genomes (KEGG) pathway classification of total changed genes, metabolism-related signaling pathways accounted for approximately 80% of the gene changes (**Supplementary Figure S4**). KEGG analysis revealed that the metabolic pathways were significantly downregulated (**Figure 4C**). To further confirm this proteomic conclusion, we used TK1 as a bait and purified TK1-interacting proteins that were identified using IP-MS, confirming successful TK1 purification (**Figure 4D**). Similarly, KEGG analysis showed that these proteins were significantly related to the development of cellular metabolic pathways (**Figure 4E**; **Supplementary Table S2**). These results indicated that TK1 binds directly to diversified metabolic enzymes, and TK1-silenced cells present with metabolic disturbance. In conclusion, TK1 was associated with metabolic reprogramming during SKCM tumorigenesis.

### Thymidine Kinase 1 Ablation Resulted in Disturbed Energy Production

**Figure 5:**
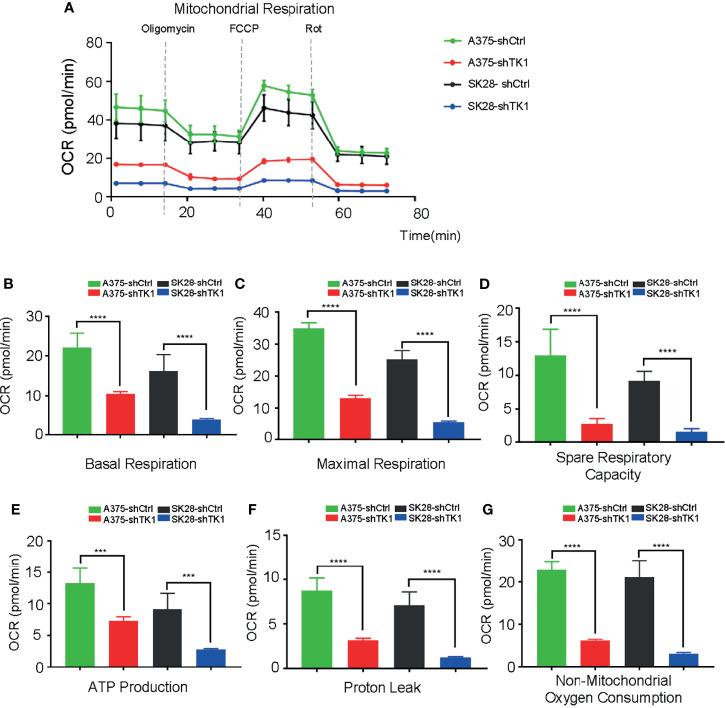
The oxygen consumption rate (OCR) was suppressed in thymidine kinase 1 (TK1) knockdown group. **(A)** The mitochondrial respiration curve of control group and TK1 downregulation group. **(B–G)** The basal respiration, maximal respiration, spare respiratory capacity, ATP production, proton leak, and non-mitochondrial oxygen consumption were inhibited in TK1 knockdown group. ***p < 0.001, ****p < 0.0001.

Supplying cells with energy consists of two processes: OxPhos and glycolysis. Cancer cells reprogram metabolism patterns to produce more energy, which supports their aggressive growth patterns (21, 22). To investigate the influence of TK1 on SKCM cell metabolism, we measured the OCR and ECAR. As a result, the mitochondrial respiration and glycolytic function of SKCM were suppressed (**Figure 5A**). A remarkable decrease in basal and maximal respiration, as well as spare respiratory capacity, was observed in shTK1 cells (**Figures 5B–D**). ATP production, the crucial procedure of cell activity, decreased with TK1 knockdown in SKCM (**Figure 5E**). Results were similar for proton leaks (**Figure 5F**). It is notable that non-mitochondrial oxygen consumption was inhibited in the shTK1 group, which indicated that oxygen consumption activity in SKCM cells was also restricted after TK1 inhibition (**Figure 5G**).

**Figure 6:**
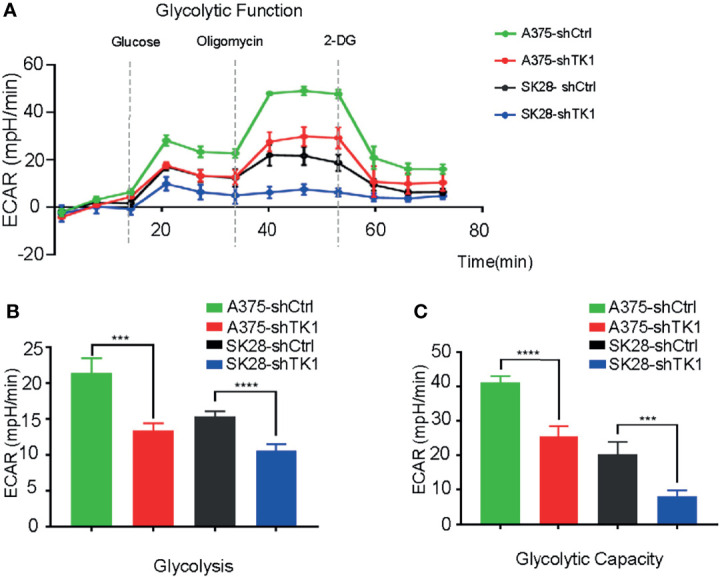
The extracellular acidification rate (ECAR) was suppressed in thymidine kinase 1 (TK1) knockdown group. **(A)** The glycolytic function curve of control group and TK1 downregulation group. **(B, C)** The glycolysis and glycolytic capacity were inhibited in TK1 knockdown group. ***p < 0.001, ****p < 0.0001.

In addition to OxPhos, enhanced glycolysis also fuels tumor progression. In our study, glycolysis and glycolytic capacity were inhibited in the shTK1 group (**Figures 6A–C**). Taken together, these data indicated that TK1 inhibition significantly reduces energy production in SKCM cells in both OxPhos and glycolysis.

### Oxidative Phosphorylation Inhibitor Attenuated the Proliferation and Migration of Skin Cutaneous Melanoma Cells

**Figure 7:**
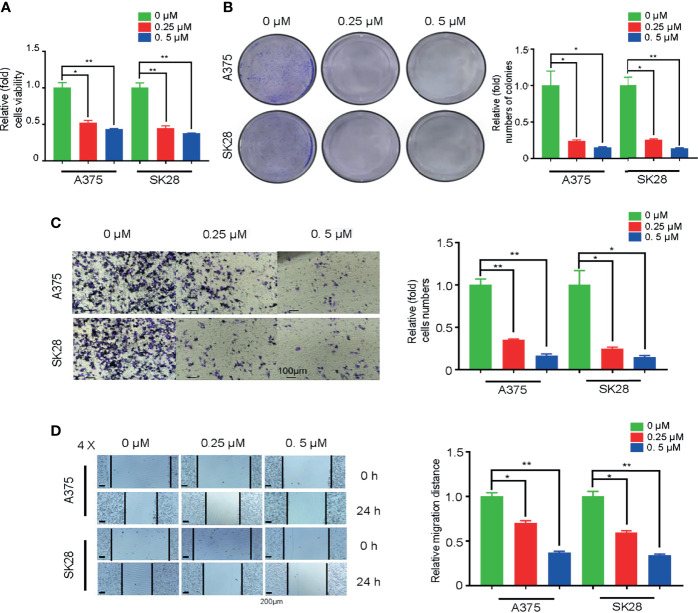
Oxidative phosphorylation (OxPhos) inhibitor attenuated proliferation and migration of skin cutaneous melanoma (SKCM) cells. **(A)** The growth ability of SKCM was attenuated after treatment with OxPhos inhibitor by Cell Counting Kit-8 (CCK-8) assay. **(B)** Colony formation assay demonstrated that SKCM formed smaller and fewer colonies after treating with 0.25 and 0.5 μM rotenone. **(C, D)** Cellular migration capacity was significantly attenuated by Transwell and wound healing assay after treatment with OxPhos inhibitor. *p < 0.05, **p < 0.01

To explore whether TK1 inhibition-induced respiratory blockade inhibits SKCM proliferation and migration, we added an OxPhos inhibitor (rotenone) to A375 and SK28 cells. Notably, after mitochondrial respiration was restrained, SKCM cells showed attenuated growth (**Figure 7A**). The colony formation assay demonstrated that SKCM formed smaller and fewer colonies after treating with 0.25 and 0.5 μM rotenone (**Figure 7B**). Moreover, cellular migration capacity was significantly attenuated, as demonstrated by Transwell (**Figure 7C**) and wound healing assay (**Figure 7D**). Taken together, these data suggest that TK1-guided respiratory inhibition plays a negative role in the tumorigenesis of SKCM cells.

## Discussion

*TK1* encodes a cytosolic enzyme that converts thymidine to deoxythymidine monophosphate (dTMP), the initial step in the biosynthesis of Deoxythymidine triphosphate (dTTP) for DNA replication. A previous study discovered that TK1 promoted pancreatic cancer cell proliferative activity *via* the E2F1-TK1-P21 axis, which focused on cell cycle regualtion (19). Besides, its overexpression in cancer types has indicated that TK1 is a promising biomarker for clinical detection (15, 17). In addition, there is an intimate association between TK1 expression and cancer prognosis. Nisman et al. (23) found that TK1 could be an individual prognostic factor for breast cancer patients. Zhang et al. (24) determined that TK1 levels could be used to supervise prognosis and surgical outcomes in primary bladder carcinoma patients. Additionally, in esophageal, cardiac, and lung carcinomas, TK1 has been found to be a reliable prognostic factor (25). Combined with the results of the current study, it is concluded that TK1 is an independent risk factor for poor prognosis. Here, we discovered that TK1 promotes SKCM malignant progression and metabolic reprogramming.

Previous studies have implicated that TK1 converts thymidine to dTMP for the regulation of thymidine metabolism. According to the RNA sequencing (RNA-seq) data, the expressions of thousands of genes involved in a wide range of intracellular activities were altered. IP-MS revealed that proteins interacting with TK1 are responsible for multiple crucial metabolic factors, including pyruvate carboxylase (PC) and glutamate dehydrogenase (GDH). Therefore, we speculate that TK1 may regulate PC activity by increasing its stability and enhancing superfluous metabolites and malignant proliferation of cancer cells. In addition, it is known that GDH participates in the Tricarboxylic acid (TCA) cycle, and it is possible that TK1 may regulate energy production through the TCA cycle to promote cancer progression (26, 27).

The incidence rate of SKCM is increasing every year, and it has become a grim clinical challenge. In recent years, considerable efforts have been made to clarify the genetic background and immunological etiology of SKCM to yield novel therapeutic effects for systemic treatment in SKCM, especially with drug resistance. Some drugs that target metabolism in cutaneous melanomas have been identified, such as HA344 (28). In addition, some gene targets have been identified. Exploiting the expression of melanogenesis-associated transcription factor target gene *CYP27A1* and inhibiting mitochondrial OxPhos suggest a strategy for *BRAF/NRAS* mutant melanoma (29). Both activated *VEGFR2 via* the pro-oncogenic *R1051Q* mutation and the *HIF-1/PDK3* bioenergetic pathway induce relevant metabolic changes in melanoma cells, serving as new targets for therapeutic intervention in melanoma (30, 31). According to our previous experimental results, we speculate that TK1 is likely to play a pivotal role in regulating multiple metabolism-related proteins that function as a switch of phosphorylation, thus influencing the intracellular metabolism pattern. Therefore, TK1 is a novel and promising therapeutic target for SKCM.

**Figure 8:**
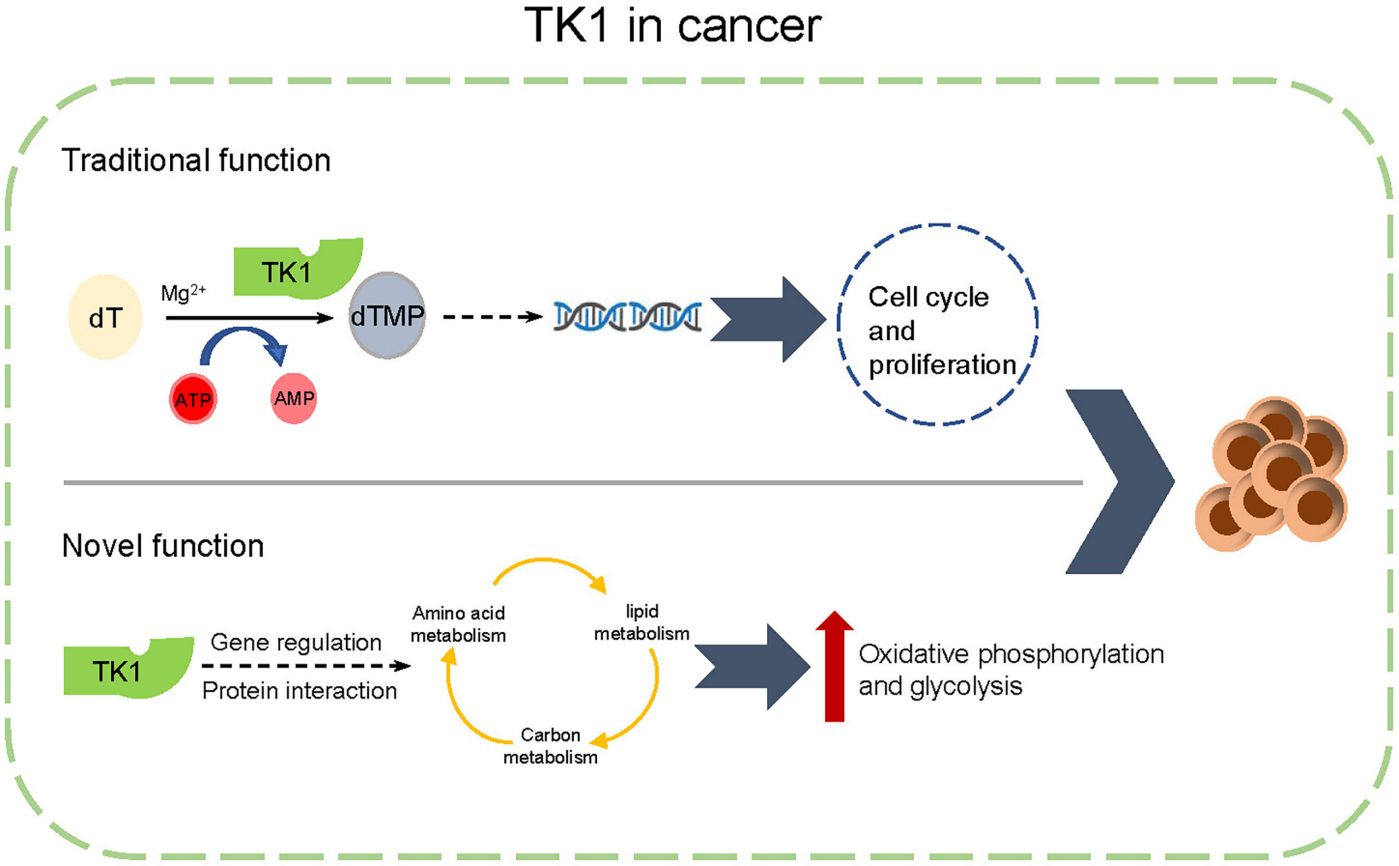
A schematic diagram of functions of thymidine kinase 1 (TK1) in skin cutaneous melanoma (SKCM).

Numerous studies have highlighted mutations in *BRAF*, *and NRAS* serves as the driving factor in the initiation and progression of SKCM (32). Herein, we have consulted the genetic background (mutation spectrum of BRAF and NRAS) of the tested cell lines (**Supplementary Table S3**). In our study, we found that TK1 inhibition attenuated both *NRAS*-mutated and *BRAF*-mutated SKCM cell lines, indicating that TK1 inhibition triggers a similar inhibitory effect in SKCM from different genetic backgrounds. Evidence has suggested that single-agent therapy does not produce a durable response in cutaneous melanoma (33). For example, although the BRAF^V600^ inhibitor vemurafenib significantly attenuated the proliferation of SKCM cells, SKCM rapidly developed drug resistance by enhancing the epithelial-to-mesenchymal transition signaling pathway (34). Moreover, a number of SKCM cases are resistant to immune checkpoint inhibitors, contributing to metabolic plasticity after exposure. Our study demonstrated that TK1 potentially regulates metabolic reprogramming in SKCM, which could give rise to an elevated metabolic adaptation for these therapies (**Figure 8**). Therefore, our conclusions suggest that TK1 inhibition might enhance the response to BRAF^V600^ inhibition as well as immune checkpoint inhibitors in SKCM cells.

In this study, we found that TK1 expression in SKCM was upregulated compared to that in matched normal tissues. Increased TK1 expression was associated with poor outcomes in SKCM. *In vitro* and *in vivo* experiments confirmed that TK1 displayed a protumorigenic role by activating the proliferation and migration of melanoma cells. Through transcriptomic and proteomic assays, we revealed that TK1 is responsible for metabolic reprogramming, which enhances mitochondrial respiration and glycolysis levels and promotes SKCM malignant progression. In conclusion, we indicate for the first time that TK1 functions as an oncogene in SKCM, which serves as a novel therapeutic target for treating SKCM.

## Conclusions

In summary, our results show that TK1 is upregulated in SKCM in comparison to that in normal tissues and cell lines. TK1 plays an oncogenic role in SKCM proliferation and migration. Furthermore, TK1 enhances metabolic reprogramming in SKCM by increasing mitochondrial respiration and glycolysis. Overall, we confirmed that TK1 can serve as a potential novel anticancer target in SKCM.

## Data Availability Statement

The raw data of RNA-seq were deposited in Sequence Read Archive in NCBI, https://www.ncbi.nlm.nih.gov/sra, SRX10950283-SRX10950285 for A375 control cells and SRX10950286-SRX10950288 for TK1-silenced A375 cells.

## Ethics Statement

The studies involving human participants were reviewed and approved by the Ethics Committee of Shanghai Jiao Tong University. Written informed consent for participation was not required for this study in accordance with the national legislation and the institutional requirements. The animal study was reviewed and approved by The Institutional Animal Care and Use Committee of the Ninth People’s Hospital.

## Author Contributions

SZ, HW, and LLi conducted all experiments and wrote the article. LNLu and HP revised this article. All authors contributed to the article and approved the submitted version.

## Funding

This project was supported by The Science and Technology Commission of Shanghai (21ZR1437400, 20DZ2270800). The funders played no role in the study design, decision to publish, or preparation of the article.

## Conflict of Interest

The authors declare that the research was conducted in the absence of any commercial or financial relationships that could be construed as a potential conflict of interest.

## Publisher’s Note

All claims expressed in this article are solely those of the authors and do not necessarily represent those of their affiliated organizations, or those of the publisher, the editors and the reviewers. Any product that may be evaluated in this article, or claim that may be made by its manufacturer, is not guaranteed or endorsed by the publisher.

## Abbreviations

TK1, thymidine kinase 1; SKCM, skin cutaneous melanoma; TCGA, The Cancer Genome Atlas; GEO, Gene Expression Omnibus; WB, Western blot; qRT-PCR, quantitative real-time PCR; RNA-seq, RNA sequencing; IP-MS, immunoprecipitation–mass spectrometry; OCR, oxygen consumption rate; ECAR, extracellular acidification rate; GAPDH, glyceraldehyde-3-phosphate dehydrogenase; PC, pyruvate carboxylase; GDH, glutamate dehydrogenase; OxPhos, Oxidative phosphorylation.
