# Supplementary material for: Thymidine Kinase 1 Drives Skin Cutaneous Melanoma Malignant Progression and Metabolic Reprogramming
Source: Front Oncol. 2022 Mar 3;12:802807. doi: 10.3389/fonc.2022.802807 (PMC8927676; doi:10.3389/fonc.2022.802807)
Supplement: Supplementary file 1 [file DataSheet_1.docx]

Supplemental files

**Thymidine kinase 1 drives melanoma malignant progression and metabolic reprogramming**

Sipeng Zuo ^1,2, #^; Huixue Wang^1,2, #^; Lin Li^1,2^;

Hui Pan ^1,2, *^; Linna Lu^1,2, *^

^1^Department of Ophthalmology, Ninth People’s Hospital, Shanghai Jiao Tong University School of Medicine, Shanghai, China.

^2^Shanghai Key Laboratory of Orbital Diseases and Ocular Oncology, Shanghai, China.

Sipeng Zuo and Huixue Wang contributed equally to this paper.

Hui Pan and Linna Lu are the co-corresponding authors.

**Institutional addresses:**

^1^Department of Ophthalmology, Ninth People’s Hospital, Shanghai Jiao Tong University School of Medicine, No 639 Zhizaoju Road, Shanghai, China.

^2^Shanghai Key Laboratory of Orbital Diseases and Ocular Oncology, Shanghai, China, No 639 Zhizaoju Road, Shanghai 200011, China.

**E-mails:**

**Hui Pan*:** xypanhui@163.com;

**Linna Lu*:** drlulinna@126.com

**Supplementary files include 5 figures and 3 Tables**

**Table legends**

**Table S1.** Primers used for qRT-PCR.

**Table S2.** Proteins interacting with TK1.

**Table S3.** Mutation of cutaneous melanoma cell lines.

**Figure legends**

**Fig S1.** Immunofluorescence on 4 nevi, 20 primary SKCMs and 10 metastatic SKCMs. Representative images are shown. The red (546nm) signal intensity was calculated in Image J. *represents p<0 .05.

**Fig S2.** The knockdown efficiency for TK1 was evaluated by qRT-PCR and WB, ** represents p<0 .01 and *** represents p<0.001.

**Fig S3.** Cell mobility of SKCM cells was determined by wound healing assay at 0, 24 h after the scratching. Each experiment was performed in triplicate. * represents p<0 .05 and ** represents p<0.01.

**Fig S4.** GO ontology classification of total changed genes with TK1 knockdown.

**Fig S5.** Unprocessed figures of western blot assays.

**Table S1. Primers used for qRT-PCR**

| Gene | | Forwards (5′-3′) | Reverse (5′-3′) | | Product |
| --- | --- | --- | --- | --- | --- |
| TK1  GAPDH | GGCACAGAGAAGGAGCAGATT  AATGGGCAGCCGTTAGGAAA | | CCACACAAAGGAGAGTTCCCA  GCCCAATACGACCAAATCAGAG | 190bp  166bp | |

**Table S2.** Proteins interacting with TK1

| Gene names | Number of Genes | Peptides TK1_IP | Sequence length | Intensity IgG | Intensity TK1_IP |
| --- | --- | --- | --- | --- | --- |
| CPS1 | 4 | 42 | 1500 | 0 | 3184800000 |
| KRT18 | 7 | 20 | 430 | 0 | 2332900000 |
| GLUD | 2 | 13 | 558 | 0 | 1252800000 |
| FGG | 5 | 10 | 445 | 0 | 1172800000 |
| FGB | 3 | 10 | 491 | 0 | 1073900000 |
| FGA | 2 | 11 | 866 | 0 | 774990000 |
| HP | 14 | 9 | 406 | 0 | 760900000 |
| ALDH2 | 3 | 10 | 517 | 0 | 748440000 |
| IDH2 | 2 | 12 | 452 | 0 | 682090000 |
| EPHX1 | 3 | 11 | 455 | 0 | 669480000 |
| ACAT1 | 4 | 10 | 427 | 0 | 657490000 |
| PC | 6 | 16 | 1178 | 0 | 580200000 |
| TK1 | 5 | 6 | 234 | 0 | 509420000 |
| IGLL | 7 | 3 | 106 | 0 | 455490000 |
| ECH1 | 6 | 6 | 328 | 0 | 398490000 |
| ALDH4A1 | 2 | 6 | 563 | 0 | 394640000 |
| TAF4B | 2 | 1 | 867 | 0 | 389790000 |
| RAP1GAP | 6 | 1 | 35 | 0 | 359720000 |
| AGXT | 1 | 9 | 392 | 0 | 341730000 |
| ABAT | 7 | 10 | 515 | 0 | 335850000 |
| PDIA3 | 1 | 10 | 505 | 0 | 327920000 |
| ACAA2 | 3 | 8 | 397 | 0 | 326620000 |
| RPSA | 4 | 7 | 263 | 0 | 324280000 |
| CYB5A | 2 | 4 | 134 | 0 | 304650000 |
| HADHB | 6 | 5 | 474 | 0 | 286810000 |
| IGKC | 1 | 3 | 107 | 0 | 275150000 |
| HSD17B6 | 3 | 4 | 317 | 0 | 221800000 |
| SPTLC | 3 | 1 | 407 | 0 | 211040000 |
| TGM2 | 4 | 6 | 687 | 0 | 210790000 |
| HSD17B4 | 11 | 8 | 711 | 0 | 209390000 |
| HMGCS2 | 1 | 4 | 508 | 0 | 196780000 |
| MGST1 | 4 | 2 | 155 | 0 | 191170000 |
| MYH9 | 7 | 14 | 1960 | 0 | 184430000 |
| CYP2E1 | 3 | 5 | 493 | 0 | 183980000 |
| PCK2 | 7 | 9 | 640 | 0 | 182820000 |
| ATP1A | 5 | 6 | 1023 | 0 | 181780000 |
| ADH4 | 5 | 4 | 391 | 0 | 180750000 |
| PRDX3 | 1 | 2 | 256 | 0 | 179240000 |
| RRBP1 | 9 | 8 | 751 | 0 | 178620000 |
| OTC | 1 | 4 | 354 | 0 | 173990000 |
| H1F0 | 1 | 2 | 194 | 0 | 172940000 |
| FLNA | 4 | 8 | 2620 | 0 | 165250000 |
| NNT | 4 | 7 | 1086 | 0 | 160290000 |
| SFN | 1 | 3 | 248 | 0 | 158890000 |
| SERPINA1 | 3 | 3 | 359 | 0 | 155710000 |
| ACADVL | 6 | 6 | 655 | 0 | 153740000 |
| ECHS1 | 1 | 4 | 290 | 0 | 151830000 |
| FN1 | 2 | 4 | 2477 | 0 | 149540000 |
| UGP2 | 9 | 4 | 508 | 0 | 147600000 |
| ACTA | 10 | 14 | 377 | 0 | 146490000 |
| RPS3A | 9 | 1 | 65 | 0 | 144920000 |
| DECR1 | 5 | 4 | 335 | 0 | 141620000 |
| C3 | 3 | 9 | 1663 | 0 | 141060000 |
| RPS13 | 3 | 5 | 151 | 0 | 135240000 |
| UTS2 | 2 | 1 | 139 | 0 | 134310000 |
| HIST1H1 | 6 | 3 | 213 | 0 | 130790000 |
| PDIA4 | 2 | 5 | 646 | 0 | 129250000 |
| IMMT | 4 | 5 | 613 | 0 | 126700000 |
| APMAP | 2 | 2 | 409 | 0 | 121840000 |
| IDH1 | 1 | 3 | 414 | 0 | 116820000 |
| RPL10;RPL10L | 7 | 1 | 91 | 0 | 114960000 |
| ACSL1 | 6 | 4 | 527 | 0 | 114890000 |
| RPN1 | 3 | 6 | 607 | 0 | 114240000 |
| CANX | 8 | 4 | 592 | 0 | 111280000 |
| HADHA | 5 | 6 | 792 | 0 | 110680000 |
| SLC25A3 | 2 | 1 | 324 | 0 | 109240000 |
| EEF1D | 22 | 4 | 281 | 0 | 105300000 |
| ADH1C | 1 | 7 | 375 | 0 | 97944000 |
| IGHG4 | 2 | 4 | 396 | 0 | 96674000 |
| CCT3 | 6 | 4 | 500 | 0 | 94705000 |
| AIFM1 | 2 | 4 | 613 | 0 | 94308000 |
| RPL15 | 6 | 2 | 133 | 0 | 93340000 |
| ETFB | 2 | 3 | 255 | 0 | 93132000 |
| RAB1 | 17 | 1 | 44 | 0 | 90350000 |
| RPL7 | 2 | 3 | 208 | 0 | 86050000 |
| GRID2 | 2 | 1 | 926 | 0 | 84713000 |
| RPS2 | 7 | 3 | 195 | 0 | 83289000 |
| TST | 2 | 3 | 297 | 0 | 82633000 |
| CYP2C9 | 1 | 2 | 490 | 0 | 82501000 |
| HNRNPA2B1 | 2 | 2 | 353 | 0 | 79847000 |
| ADH1A | 1 | 6 | 375 | 0 | 78286000 |
| NONO;SFPQ | 4 | 1 | 207 | 0 | 78032000 |
| RPL12 | 1 | 3 | 165 | 0 | 77776000 |
| YWHAB | 5 | 4 | 246 | 0 | 77622000 |
| AIM1 | 3 | 4 | 1723 | 0 | 76191000 |
| ACSM2 | 8 | 4 | 498 | 0 | 76103000 |
| ALDH6A1 | 2 | 4 | 535 | 0 | 74699000 |
| PDIA6 | 1 | 2 | 440 | 0 | 74469000 |
| SORD | 3 | 2 | 357 | 0 | 72557000 |
| ALDH1A1 | 1 | 3 | 501 | 0 | 72066000 |
| HLA-A | 50 | 3 | 362 | 0 | 69480000 |
| APOA1 | 2 | 2 | 245 | 0 | 69474000 |
| ILF2 | 3 | 2 | 390 | 0 | 68235000 |
| FH | 1 | 3 | 510 | 0 | 67322000 |
| COL6A3 | 2 | 5 | 2569 | 0 | 66944000 |
| MT-CO2 | 1 | 1 | 227 | 0 | 66241000 |
| CFL | 7 | 1 | 79 | 0 | 65900000 |
| COL6A1 | 2 | 2 | 1026 | 0 | 65396000 |
| RPL3;RPL3L | 3 | 1 | 351 | 0 | 65222000 |
| LAP3 | 2 | 4 | 519 | 0 | 62568000 |
| ATP1A2;ATP4A | 6 | 3 | 1009 | 0 | 62324000 |
| SFXN1 | 5 | 3 | 322 | 0 | 61755000 |
| RDH16 | 1 | 3 | 317 | 0 | 61098000 |
| RPL18A | 5 | 3 | 141 | 0 | 60943000 |
| RPL35 | 2 | 1 | 96 | 0 | 60559000 |
| RPS25 | 1 | 2 | 125 | 0 | 59513000 |
| RPS11 | 2 | 1 | 118 | 0 | 59496000 |
| PRKDC | 1 | 5 | 4128 | 0 | 57985000 |
| CS | 15 | 2 | 400 | 0 | 57855000 |
| PRDX4 | 3 | 5 | 161 | 0 | 57147000 |
| RAB6;RAB39A | 6 | 1 | 49 | 0 | 55038000 |
| ACADS | 2 | 2 | 408 | 0 | 54250000 |
| HMGCL | 2 | 3 | 325 | 0 | 53153000 |
| HBD | 4 | 7 | 147 | 0 | 52397000 |
| TF | 5 | 4 | 698 | 0 | 51849000 |
| RPL13 | 4 | 2 | 211 | 0 | 50685000 |
| SDHA | 4 | 2 | 356 | 0 | 50598000 |
| GNB | 16 | 1 | 79 | 0 | 49847000 |
| SND1 | 2 | 1 | 231 | 0 | 47625000 |
| IGHG3 | 3 | 7 | 446 | 0 | 47533000 |
| DBN1 | 3 | 2 | 124 | 0 | 47044000 |
| TMEM132B | 1 | 1 | 1078 | 0 | 46052000 |
| MAOB;MAOA | 2 | 2 | 520 | 0 | 45978000 |
| ACADM | 4 | 2 | 385 | 0 | 45016000 |
| CTSA | 3 | 2 | 498 | 0 | 44281000 |
| RPL10A | 1 | 1 | 217 | 0 | 43527000 |
| COL6A2 | 2 | 2 | 1019 | 0 | 42722000 |
| RPS18 | 2 | 2 | 152 | 0 | 42223000 |
| GLB1 | 3 | 3 | 677 | 0 | 42184000 |
| CAPZA2;CAPZA1 | 5 | 1 | 146 | 0 | 41651000 |
| RPL27 | 3 | 1 | 38 | 0 | 41530000 |
| DDX39B;DDX39A | 23 | 2 | 428 | 0 | 40939000 |
| ACTN1 | 8 | 7 | 822 | 0 | 40225000 |
| ARF4 | 4 | 2 | 153 | 0 | 40157000 |
| RPS7 | 3 | 1 | 169 | 0 | 38834000 |
| ACSL5 | 1 | 2 | 683 | 0 | 38562000 |
| TUBA1 | 27 | 14 | 449 | 0 | 37697000 |
| HSPE1 | 4 | 2 | 102 | 0 | 36371000 |
| H3F3 | 10 | 3 | 92 | 0 | 35669000 |
| PDIA3 | 1 | 4 | 123 | 0 | 35643000 |
| PSMD2 | 2 | 3 | 908 | 0 | 34867000 |
| PCBP2 | 7 | 3 | 160 | 0 | 34258000 |
| HNRNPU | 12 | 3 | 728 | 0 | 33741000 |
| 2-Mar | 2 | 1 | 94 | 0 | 33659000 |
| UQCRC2 | 2 | 2 | 412 | 0 | 33028000 |
| SERPINA3 | 3 | 2 | 423 | 0 | 33006000 |
| RPS10 | 7 | 3 | 121 | 0 | 32626000 |
| UQCRC1 | 1 | 1 | 480 | 0 | 32486000 |
| GNAT | 22 | 1 | 54 | 0 | 32187000 |
| CYB5R3 | 2 | 1 | 147 | 0 | 32171000 |
| ATP2A | 3 | 2 | 933 | 0 | 31905000 |
| BDH1 | 7 | 2 | 129 | 0 | 31674000 |
| UGDH | 7 | 2 | 494 | 0 | 31574000 |
| RPL4 | 2 | 1 | 333 | 0 | 31303000 |
| TXNDC5 | 1 | 2 | 432 | 0 | 31071000 |
| ATP5F1 | 2 | 1 | 195 | 0 | 30619000 |
| GDI2;GDI1 | 4 | 1 | 203 | 0 | 30488000 |
| RAD23 | 7 | 2 | 409 | 0 | 30447000 |
| TUFM | 1 | 1 | 452 | 0 | 30335000 |
| HSD17B10 | 2 | 2 | 261 | 0 | 29959000 |
| EHHADH | 1 | 1 | 723 | 0 | 29848000 |
| GATM | 4 | 2 | 423 | 0 | 29494000 |
| OSMR | 1 | 1 | 979 | 0 | 29427000 |
| HLA-DRA | 9 | 1 | 190 | 0 | 29273000 |
| EIF3A | 1 | 3 | 1382 | 0 | 29075000 |
| RPS19 | 2 | 1 | 71 | 0 | 27964000 |
| IGHG2 | 2 | 4 | 395 | 0 | 27821000 |
| METTL7A | 3 | 1 | 180 | 0 | 27773000 |
| STOM | 2 | 2 | 288 | 0 | 27689000 |
| SHMT1 | 1 | 3 | 483 | 0 | 27169000 |
| RPL14 | 2 | 1 | 124 | 0 | 26900000 |
| IQGAP1 | 2 | 3 | 1191 | 0 | 26804000 |
| SEC23A;SEC23B | 9 | 1 | 93 | 0 | 26524000 |
| IGKV3D-11 | 2 | 1 | 115 | 0 | 26265000 |
| HNRNPC | 21 | 1 | 54 | 0 | 26120000 |
| FMO3 | 1 | 1 | 532 | 0 | 26062000 |
| ARHGDIA | 6 | 1 | 92 | 0 | 25474000 |
| SRSF3 | 2 | 1 | 95 | 0 | 25276000 |
| SLC25A11 | 2 | 2 | 296 | 0 | 25084000 |
| CCT2 | 3 | 2 | 416 | 0 | 24956000 |
| TMED9 | 1 | 1 | 235 | 0 | 24863000 |
| PGRMC1 | 1 | 2 | 195 | 0 | 24683000 |
| NEFL | 1 | 3 | 543 | 0 | 24587000 |
| CSE1L | 1 | 2 | 971 | 0 | 24267000 |
| DARS | 4 | 2 | 501 | 0 | 24071000 |
| RARS | 1 | 3 | 660 | 0 | 23635000 |
| APCS | 1 | 2 | 223 | 0 | 23467000 |
| ACO2 | 2 | 1 | 805 | 0 | 23273000 |
| BCAP31 | 4 | 1 | 182 | 0 | 22445000 |
| COL1A2 | 2 | 2 | 1364 | 0 | 22377000 |
| GART | 1 | 2 | 1010 | 0 | 22305000 |
| CHCHD3 | 3 | 1 | 241 | 0 | 22222000 |
| HSD11B1 | 2 | 1 | 278 | 0 | 21890000 |
| CLIC1 | 1 | 2 | 241 | 0 | 21878000 |
| MGST2 | 1 | 1 | 147 | 0 | 21824000 |
| CTNNA | 4 | 1 | 129 | 0 | 21730000 |
| GSTK1 | 2 | 1 | 190 | 0 | 21313000 |
| PSMA1 | 2 | 1 | 238 | 0 | 21227000 |
| SLC27A5 | 1 | 1 | 690 | 0 | 21168000 |
| HARS | 5 | 1 | 288 | 0 | 21128000 |
| HNRNPR;  SYNCRIP | 5 | 2 | 633 | 0 | 20890000 |
| IARS | 3 | 1 | 1152 | 0 | 20843000 |
| PSMB2 | 2 | 2 | 84 | 0 | 20681000 |
| ARPC4 | 6 | 2 | 77 | 0 | 20402000 |
| ATP5O | 3 | 1 | 155 | 0 | 20361000 |
| LONP1 | 4 | 2 | 829 | 0 | 20148000 |
| RPL13 | 4 | 1 | 142 | 0 | 19397000 |
| H6PD | 2 | 1 | 802 | 0 | 19292000 |
| HNRNPM | 7 | 2 | 352 | 0 | 19135000 |
| GRN | 4 | 1 | 110 | 0 | 19077000 |
| CBR3 | 1 | 1 | 277 | 0 | 18960000 |
| EIF3L | 6 | 2 | 607 | 0 | 18940000 |
| FTH1 | 6 | 1 | 50 | 0 | 18936000 |
| PCCA | 3 | 1 | 139 | 0 | 18863000 |
| TUBB3 | 2 | 7 | 797 | 0 | 18623000 |
| CDC42 | 1 | 1 | 191 | 0 | 18482000 |
| 11-Sep | 3 | 1 | 425 | 0 | 18294000 |
| FASN | 2 | 2 | 2509 | 0 | 18217000 |
| NACA | 7 | 1 | 71 | 0 | 18141000 |
| SLC25A13 | 1 | 2 | 675 | 0 | 17673000 |
| CAND1 | 3 | 2 | 1230 | 0 | 17634000 |
| SPTAN1 | 3 | 4 | 2457 | 0 | 17499000 |
| ECI1;DCI | 3 | 2 | 224 | 0 | 17400000 |
| ALDH5A1 | 2 | 1 | 507 | 0 | 17345000 |
| APOH | 2 | 1 | 181 | 0 | 17130000 |
| RPN2 | 3 | 1 | 166 | 0 | 16937000 |
| PSMA5 | 1 | 1 | 241 | 0 | 16773000 |
| SSR1 | 6 | 1 | 265 | 0 | 16724000 |
| DEFA3;DEFA1 | 2 | 1 | 94 | 0 | 16723000 |
| AK3 | 1 | 1 | 227 | 0 | 16541000 |
| HSDL2 | 1 | 2 | 418 | 0 | 16468000 |
| DAD1 | 4 | 1 | 65 | 0 | 16404000 |
| SEC61B | 2 | 2 | 96 | 0 | 16337000 |
| WDR1 | 1 | 1 | 606 | 0 | 15897000 |
| EIF3G | 5 | 2 | 289 | 0 | 15526000 |
| LGALS4 | 1 | 1 | 323 | 0 | 15523000 |
| SDHB | 3 | 1 | 123 | 0 | 15477000 |
| CYP4F | 6 | 2 | 199 | 0 | 15239000 |
| HIBADH | 1 | 1 | 336 | 0 | 15079000 |
| CNDP2 | 13 | 2 | 475 | 0 | 15030000 |
| DHX9 | 1 | 2 | 1270 | 0 | 14848000 |
| RBBP4;RBBP7 | 8 | 1 | 167 | 0 | 14772000 |
| SCAMP3 | 1 | 1 | 347 | 0 | 14717000 |
| EIF3B | 2 | 1 | 302 | 0 | 14708000 |
| BHMT | 2 | 1 | 253 | 0 | 14526000 |
| RAB7A | 4 | 1 | 116 | 0 | 13843000 |
| IPO7 | 1 | 1 | 1038 | 0 | 13765000 |
| DDB1 | 2 | 1 | 1092 | 0 | 13607000 |
| OSTC | 2 | 1 | 83 | 0 | 13439000 |
| AKR1D1 | 1 | 1 | 326 | 0 | 13313000 |
| NCBP2-AS2 | 1 | 1 | 99 | 0 | 13300000 |
| COL1A1 | 1 | 1 | 1464 | 0 | 13198000 |
| SUCLG2 | 2 | 1 | 379 | 0 | 12877000 |
| COL4A | 3 | 1 | 386 | 0 | 12805000 |
| PGAM2 | 3 | 1 | 253 | 0 | 12402000 |
| PSMC5 | 2 | 1 | 263 | 0 | 12311000 |
| USMG5 | 1 | 1 | 58 | 0 | 12006000 |
| COPA | 6 | 2 | 1024 | 0 | 11766000 |
| ALDH7A1 | 12 | 1 | 164 | 0 | 11619000 |
| ABHD14B | 3 | 1 | 188 | 0 | 11572000 |
| FLNB;FLNC | 5 | 5 | 2602 | 0 | 11532000 |
| MYO5B | 2 | 1 | 965 | 0 | 11320000 |
| PCYOX1 | 3 | 1 | 209 | 0 | 11211000 |
| CYP2C8 | 4 | 3 | 307 | 0 | 11119000 |
| UGT2B7 | 3 | 1 | 335 | 0 | 10962000 |
| SCCPDH | 1 | 1 | 429 | 0 | 10935000 |
| FLOT2 | 4 | 1 | 249 | 0 | 10650000 |
| RTN4 | 2 | 1 | 345 | 0 | 10476000 |
| SERPING1 | 3 | 1 | 463 | 0 | 10050000 |
| COPG2;COPG1 | 2 | 2 | 871 | 0 | 10042000 |
| LMAN2 | 4 | 1 | 191 | 0 | 9811400 |
| PPP1C | 11 | 1 | 125 | 0 | 9709700 |
| ALDH1A3 | 2 | 1 | 405 | 0 | 9696600 |
| LMNB1 | 1 | 3 | 586 | 0 | 9571600 |
| RPLP1 | 1 | 1 | 114 | 0 | 9528200 |
| HNRNPD | 5 | 1 | 111 | 0 | 9458000 |
| HKDC1;HK1 | 2 | 1 | 917 | 0 | 9438400 |
| PSMD5 | 2 | 2 | 504 | 0 | 9299100 |
| TMED4 | 1 | 1 | 227 | 0 | 9195500 |
| SQRDL | 2 | 1 | 124 | 0 | 8862700 |
| FBP1 | 2 | 1 | 273 | 0 | 8834800 |
| GRIA2 | 2 | 1 | 730 | 0 | 8736100 |
| AKR7A3 | 1 | 1 | 331 | 0 | 8719500 |
| PTPN1 | 2 | 2 | 362 | 0 | 8671100 |
| TPT1;TPT1P8 | 6 | 1 | 160 | 0 | 8555300 |
| AOX1 | 1 | 2 | 1338 | 0 | 8321700 |
| RPL23 | 4 | 1 | 91 | 0 | 8275100 |
| ATP5J2 | 4 | 1 | 54 | 0 | 8252100 |
| NASP | 1 | 1 | 788 | 0 | 8166300 |
| NPEPPS | 2 | 1 | 915 | 0 | 8054700 |
| UGT2B15 | 2 | 1 | 530 | 0 | 7725300 |
| PSMC6 | 4 | 1 | 194 | 0 | 7265000 |
| SPCS2 | 4 | 1 | 157 | 0 | 7218900 |
| KIN27;PRKACA | 3 | 1 | 207 | 0 | 7176500 |
| AGL | 1 | 1 | 1532 | 0 | 7155100 |
| TRIP11 | 2 | 1 | 1695 | 0 | 7091500 |
| PA2G4 | 2 | 2 | 284 | 0 | 6837800 |
| SLC26A2 | 1 | 1 | 739 | 0 | 6778700 |
| MOGS | 3 | 1 | 562 | 0 | 6512700 |
| HNRNPF | 1 | 1 | 415 | 0 | 6480400 |
| STOML2 | 1 | 1 | 356 | 0 | 6442200 |
| DDOST | 2 | 1 | 439 | 0 | 6313600 |
| RUVBL1 | 5 | 2 | 315 | 0 | 6280700 |
| ARCN1 | 3 | 1 | 189 | 0 | 6212300 |
| RPL29 | 2 | 1 | 167 | 0 | 6189900 |
| SF3B1 | 1 | 1 | 1304 | 0 | 5930900 |
| RHOB | 1 | 2 | 196 | 0 | 5872700 |
| CSRP1 | 4 | 1 | 153 | 0 | 5765100 |
| PAICS | 3 | 1 | 333 | 0 | 5738000 |
| PSAT1 | 1 | 1 | 370 | 0 | 5675900 |
| HSD17B12 | 2 | 1 | 276 | 0 | 5185300 |
| BLVRB | 5 | 1 | 121 | 0 | 5123400 |
| PSMD3 | 2 | 1 | 117 | 0 | 4944500 |
| RUVBL2 | 2 | 1 | 354 | 0 | 4933400 |
| AIMP2 | 2 | 1 | 242 | 0 | 4932800 |
| SAMM50 | 1 | 1 | 469 | 0 | 4788200 |
| CD81 | 7 | 1 | 165 | 0 | 4787700 |
| ALDH1B1 | 1 | 1 | 517 | 0 | 4347500 |
| SRP14 | 2 | 1 | 115 | 0 | 4121500 |
| ABCD3 | 1 | 1 | 659 | 0 | 3933500 |
| NDUFS8 | 5 | 1 | 110 | 0 | 3841100 |
| ACTG1 | 7 | 20 | 375 | 0 | 3337200 |
| ORM1 | 1 | 1 | 201 | 0 | 3316100 |
| SURF4 | 2 | 1 | 186 | 0 | 3105100 |
| NPM3 | 1 | 1 | 178 | 0 | 2864300 |
| ANXA4 | 2 | 1 | 299 | 0 | 2705000 |
| LETM1 | 1 | 1 | 739 | 0 | 2488400 |

**Table S3** Mutation of cutaneous melanoma cell lines.

| **Cell line** | A375 | SKMEL5 | | SKMEL28 | |
| --- | --- | --- | --- | --- | --- |
| **Mutation** | *BRAF^V600E^/NRAS^Q61K^* | | *BRAF^V600E^* | | *BRAF^V600E^* |
| **Reference** | Jana Jandova et.al^1^ | | Xu Zhang et.al^2^ | | Xu Zhang et.al^2^ |
| **Cell line** | LOXIMVI | | M14 | | M21 |
| **Mutation** | *BRAF^V600E^* | | *BRAF^V600E^* | | *BRAF^V600E^* |
| **Reference** | Eslam M H Ali et.al^3^ | | Alexandra Frazao et.al^4^ | | Hui Jin et.al^5^ |

**References**

1. Jandova, J. & Wondrak, G.T. Vemurafenib Drives Epithelial-to-Mesenchymal Transition Gene Expression in BRAF Inhibitor‒Resistant BRAF(V600E)/NRAS(Q61K) Melanoma Enhancing Tumor Growth and Metastasis in a Bioluminescent Murine Model. *The Journal of investigative dermatology* (2021).

2. Zhang, X.*, et al.* CX-F9, a novel RSK2 inhibitor, suppresses cutaneous melanoma cells proliferation and metastasis through regulating autophagy. *Biochemical pharmacology* **168**, 14-25 (2019).

3. Ali, E.M.H.*, et al.* Design, synthesis, biological evaluation, and docking studies of novel (imidazol-5-yl)pyrimidine-based derivatives as dual BRAF(V600E)/p38α inhibitors. *European journal of medicinal chemistry* **215**, 113277 (2021).

4. Frazao, A.*, et al.* BRAF inhibitor resistance of melanoma cells triggers increased susceptibility to natural killer cell-mediated lysis. *Journal for immunotherapy of cancer* **8**(2020).

5. Jin, H., Sun, Y., Wang, S. & Cheng, X. Matrine activates PTEN to induce growth inhibition and apoptosis in V600EBRAF harboring melanoma cells. *International journal of molecular sciences* **14**, 16040-16057 (2013).
