# Supplementary figures and images for: Thymidine Kinase 1 Drives Skin Cutaneous Melanoma Malignant Progression and Metabolic Reprogramming
Source: Front Oncol. 2022 Mar 3;12:802807. doi: 10.3389/fonc.2022.802807 (PMC8927676; doi:10.3389/fonc.2022.802807)

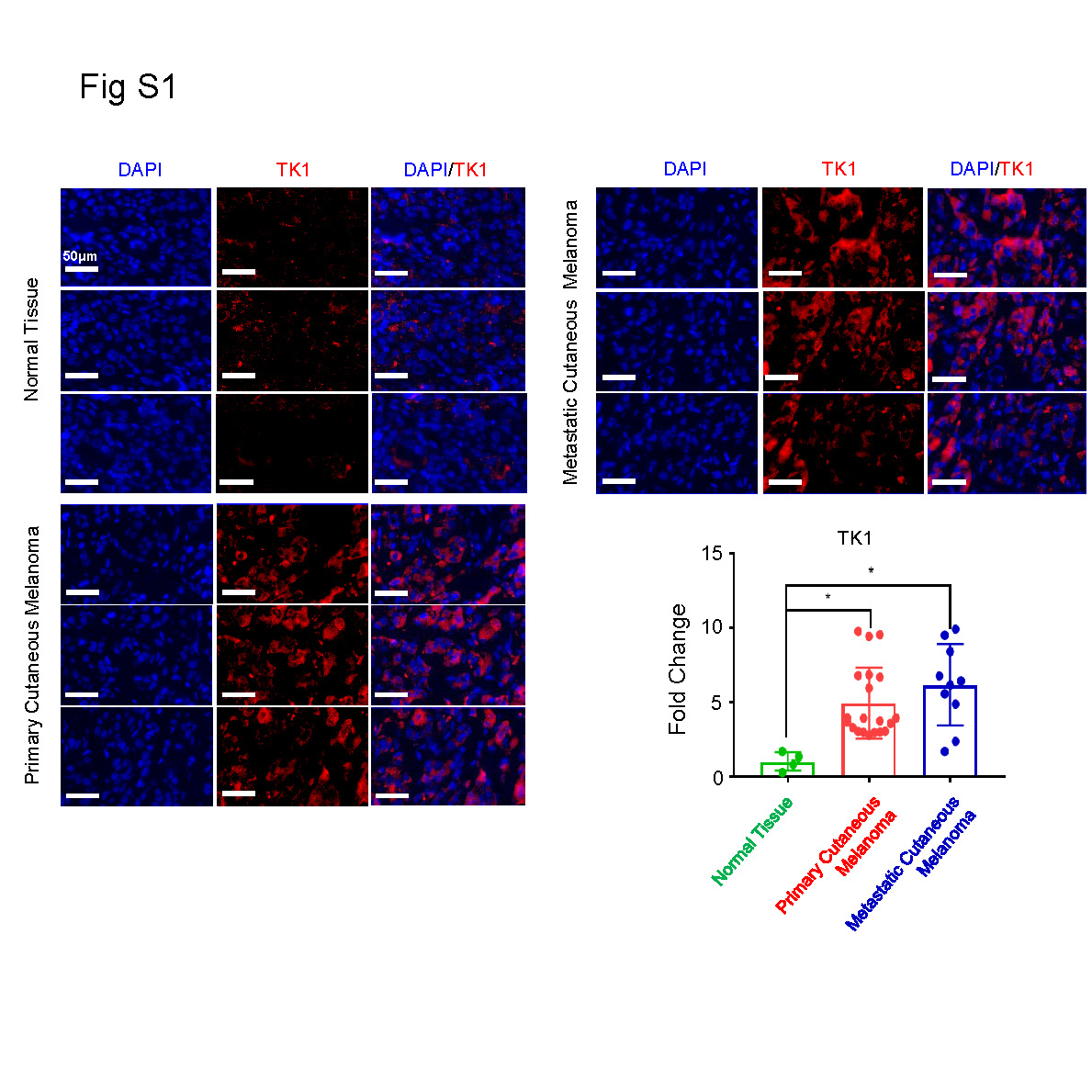

Supplement: Supplementary file 2 [file Image_1.jpeg]

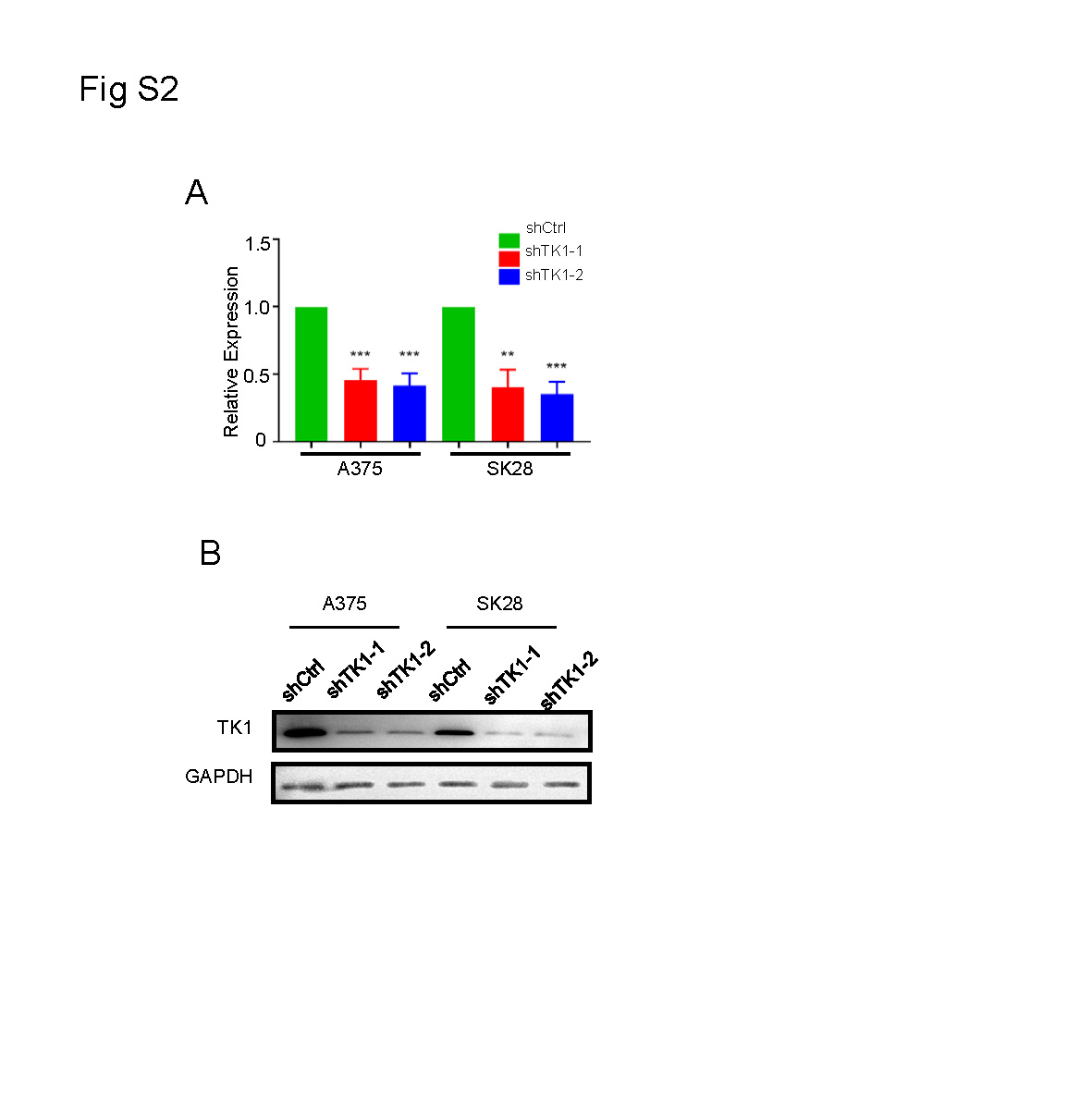

Supplement: Supplementary file 3 [file Image_2.jpeg]

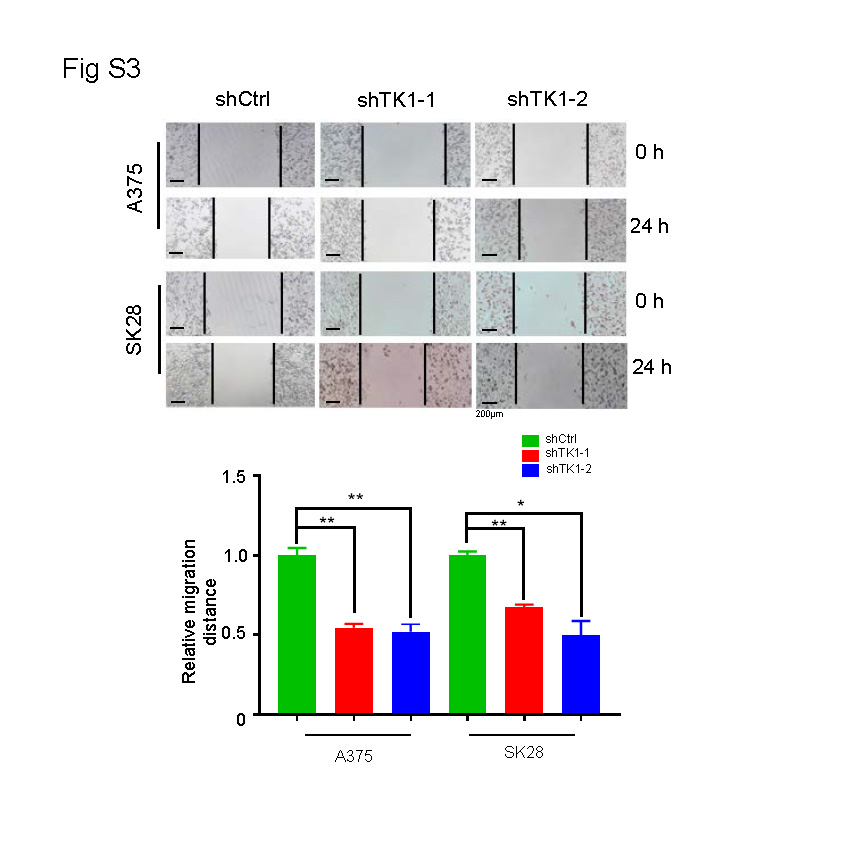

Supplement: Supplementary file 4 [file Image_3.jpeg]

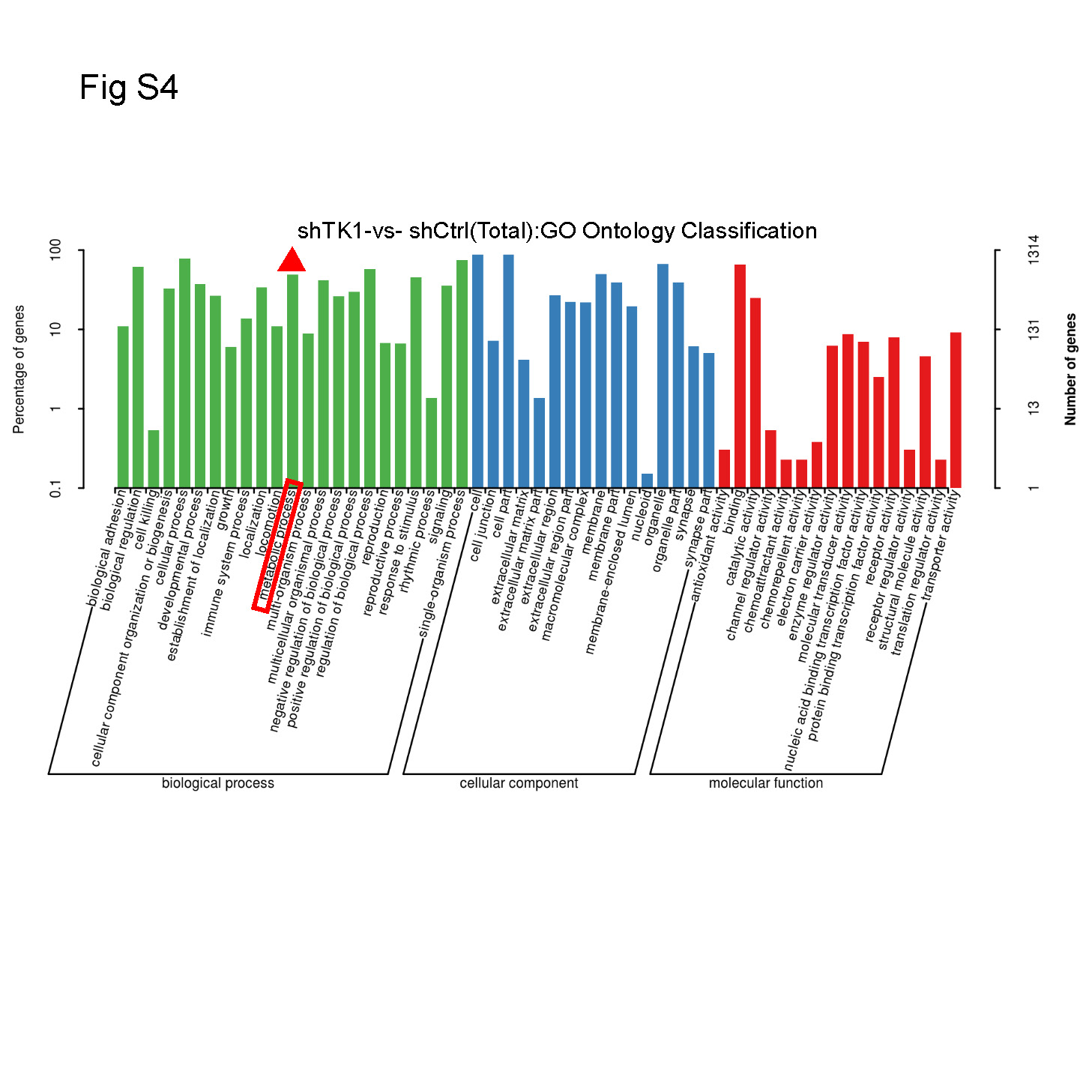

Supplement: Supplementary file 5 [file Image_4.jpeg]

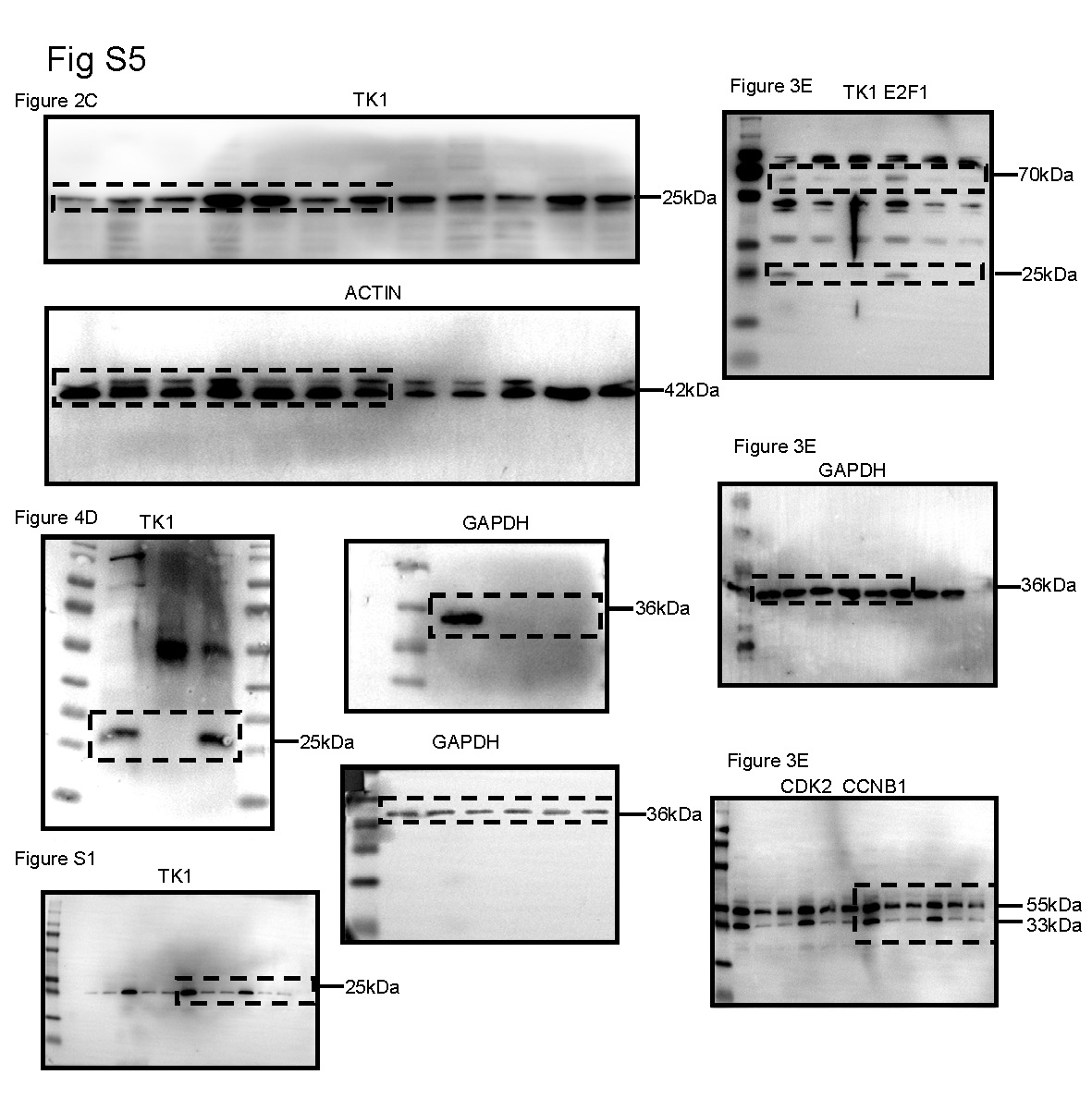

Supplement: Supplementary file 6 [file Image_5.jpeg]
